# Human life expectancy and season of birth in Taiwan: A retrospective cohort study

**DOI:** 10.1007/s00114-024-01933-5

**Published:** 2024-10-07

**Authors:** Tsutomu Nishimura, Eiji Nakatani, Mei-Chen Lin, Hiroyuki Yamauchi, Masanori Fukushima, Chung Y. Hsu

**Affiliations:** 1 Institute for Advancement of Clinical and Translational Science (iACT), Kyoto University Hospital, Kyoto, 606-8507 Japan; 2 Department of Biostatistics and Health Data Science, Graduate School of Medical Science, Nagoya City University, Nagoya, 467-8601 Japan; 3 Management Office for Health Data, China Medical University Hospital, Taichung, 404 Taiwan; 4 Statistical Analysis Division, Earthquake Prediction Research Center, Tokyo, 103-0014 Japan; 5 Intelligent Health Promotion Division, Learning Health Society Institute, Nagoya, 451-6005 Japan; 6 Graduate Institute of Biomedical Sciences, China Medical University, Taichung, 404 Taiwan

**Keywords:** Birth month, Life expectancy, Environmental factors

## Abstract

Prior research has indicated a correlation between the birth season and life expectancy; however, many of these studies did not sufficiently account for comorbidities. In this comprehensive investigation, we aimed to meticulously explore the association between the birth month and life expectancy, giving due consideration to comorbidities. We used a robust dataset derived from Taiwan’s National Health Insurance Research Database (2000–2013), which allowed us to conduct a thorough examination. We divided our participants into four groups based on their season of birth: spring, summer, autumn, and winter. Propensity score matching was used to ensure an equitable distribution of demographic and clinical characteristics across the groups. Propensity scores were computed using logistic regression. Our model incorporated a broad range of demographic factors and comorbidities, providing rigorous adjustment for potential confounders. Our findings revealed a significantly increased risk of all-cause mortality among individuals born in spring, even after stringent adjustment for demographic factors and comorbidities. People born in spring demonstrated a 1.05-fold increase in the risk of all-cause mortality, with a hazard ratio of 1.05 and a 95% confidence interval of 1.01–1.09. Our study provides compelling evidence that helps understand the potential long-term impacts of a person’s birth season, which acts as a proxy for pregnancy / early-life environmental exposure, on life expectancy. These findings underscore the crucial need for additional research to illuminate the underlying biological and environmental mechanisms linking the birth season and lifespan of a person. The elucidation of these links could guide the development of innovative health promotion and disease prevention strategies that are tailored to an individual’s birth season.

## Introduction

Studies have reported a correlation between the birth month and the life expectancy of a person (Moore et al. 1997; Doblhammer and Vaupel 2001; Vaiserman et al. 2002; Lerchl 2004; Gavrilov and Gavrilova 2011; Ueda et al. 2013; Cozzani and Barban 2023). Adults aged over 50 years in the northern hemisphere who were born in autumn have a longer life expectancy than those born in spring (Doblhammer and Vaupel 2001; Lerchl 2004; Ueda et al. 2013). In two countries in the northern hemisphere (Austria and Denmark), people born in the fall (October–December) live longer than those born in the spring (April–June), and Australian data show that in the Southern hemisphere, the pattern shifts by six months (Doblhammer and Vaupel 2001). This observation is explained by the fact that the early-life environment influences a person’s susceptibility to infectious and chronic diseases in adulthood (Doblhammer and Vaupel 2001). A nationwide study conducted in Sweden showed that the month of birth was a significant predictor of the mortality risk for people aged > 30 years, >50 to 80 years, and > 80 years. For ages >30 and >50 to 80 years, people born in November had the lowest mortality rates, whereas those born in spring or during summer had the highest mortality rates (Ueda et al. 2013). The possible mechanism involved is vitamin D deficiency because skin exposure to ultraviolet (UVB) radiation is a major determinant of vitamin D levels in humans, and the prenatal period is a critical moment when vitamin D deficiency predisposes the fetus to adult diseases (Ueda et al. 2013).

However, in previous studies, the effects of comorbidities were not adjusted for, which could significantly alter the relationship between the birth month and life expectancy. For instance, cardiovascular disease, a common comorbidity in older adults, could disproportionately affect those born in certain months (Zhang et al. 2019). It has also been reported that the incidence of various diseases—such as amyotrophic lateral sclerosis, alcohol abuse, asthma, Down’s syndrome, childhood diabetes mellitus, Crohn’s disease, Hodgkin’s disease, glaucoma, multiple sclerosis, autism, bipolar disorder, personality disorders, eating disorders, social anxiety disorder, neuroses, schizoaffective disorder, suicidal tendencies, Alzheimer’s disease, mental retardation, epilepsy, motor neuron disease, narcolepsy, and Parkinson’s disease—varies depending on the individual’s birth month (Foster and Roenneberg 2008). For instance, individuals born in winter have been found to have a higher incidence of schizophrenia and bipolar disorder, two conditions that are known to influence a person’s life expectancy (Foster and Roenneberg 2008).

In the present study, after adjusting for comorbidities, we investigated whether a birth month–life expectancy correlation would exist; we carried out the aforementioned analysis using health insurance data from Taiwan, as this data set provides a comprehensive view of the population’s health status and includes detailed information on each person’s birth month and life expectancy.

We aimed to determine whether the correlation between the birth month and life expectancy would persist after adjusting for comorbidities. We used hazard ratios (HRs) and their corresponding 95% confidence intervals (95% CIs) to evaluate the prognostic risk, with a focus on the potential impact of comorbidities on this relationship. To the best of our knowledge, this is the first study in which such factors are adjusted for when determining the association that exists between the birth month and life expectancy. The findings of this study could provide new insights into the complex interplay of these variables.

## Materials and methods

### Source of the analyzed data

The Taiwanese government has implemented and manages its renowned National Health Insurance Program to foster and enhance the health of Taiwan residents. The proportion of insured people in the Taiwanese population currently exceeds 99%. Established in 1995, its widely used National Health Insurance Research Database (NHIRD) is updated once a year. It contains information on each insurant concerning outpatient visits, hospitalizations, prescriptions, medical treatments, and other medical services received. We performed our analyses by employing a subset of the above database, namely the Longitudinal Health Insurance Database 2000, comprising deidentified or encrypted NHIRD data of one million individuals for use in research. The coding of the database’s diagnosis records is based on the International Classification of Disease, Ninth Revision, Clinical Modification (ICD-9-CM). Our study was approved by China Medical University and Hospital’s Research Ethics Committee (approval number: CMUH-104-REC2-115-R3).

### Study population

Our study included patients with outpatient or hospitalization records of 2000–2013, with January 1, 2000 serving as the index date (accessed 2015–11-13). We classified the patients into four groups according to the season during which they were born (spring, summer, fall, and winter, corresponding to March–May, June–August, September–November, and December–February, respectively). We defined the event occurrence day as the date of the patient’s death due to all-cause mortality. We excluded patients without follow-up and those missing data on sex or age. We also considered in our examination the following comorbidities (with their corresponding ICD-9-CM codes, if any, provided in parentheses): coronary artery disease (410–414, A270, and A279), cancer, hypertension (401–405, A260, and A269), diabetes mellitus (250), sleep disorders (307.4 and 780.5), dialysis (order codes: 58001C, 58002C, 58007C, 58014C, 58017C, 58018C, 58029C, and 58030B), depression (296.2, 296.3, 296.82, 300.4, 309.0, 309.1, 309.28, and 311), atopic dermatitis (691.8), head injury (310.2, 800, 801, 803, 804, 850–854, and 959.01), stroke (430–438), mental disorders (290–319 and A21), neurological diseases (293, 348.1, 348.3, 780.01, 780.09, and 89.14), chronic kidney disease (580–589 and A350), allergy rhinitis (477), asthma (493), allergy conjunctivitis (372.05, 372.10, and 372.14), dementia (290, 294.1, and 331.0), chronic obstructive pulmonary disease (COPD) (490–492, 494, and 496), hyperlipidemia (272 and A182), and osteoporosis (733.0). Comorbidities were recognized as a minimum of two outpatient visits or one hospitalization for the condition before the index date.

### Statistical analysis

In the present study, we matched the propensity scores for each pair among the four groups. Logistic regression was employed to calculate propensity scores, and the probability (prognostic score) was estimated to match with corresponding groups. The distributions determined for age groups, sex, and comorbidities in each cohort are presented as frequencies and percentages. Through standardized mean difference (SMD) calculations, we carried out comparisons in each two-pair established group and set winter as the reference group. An SMD of ≤ 0.1 indicated a negligible difference. For each patient, we defined the follow-up period (expressed in person-years) as the period from the index date to the patient’s death, loss to follow-up, date of the last follow-up, or December 31, 2013 for patients without event occurrence. We estimated HRs and 95% CIs by using proportional hazard models. We adjusted for comorbidities, sex, occupation, age, urbanization level, and income, after which we derived the corresponding adjusted HRs by applying a multivariable Cox proportional hazards model. In addition, the study evaluated the birth season–all-cause mortality associations through multivariable stratified analyses. A Kaplan–Meier plot was established to reflect the cumulative incidence among the four groups. We assessed the mentioned incidence by performing a log-rank test. We performed the aforementioned statistical analyses using SAS version 9.4 (SAS Institute Inc., Cary, NC, USA). We also used R software (R Foundation, Vienna, Austria) to derive the mentioned Kaplan–Meier plot. Based on tailed tests, we established the statistical significance level as *p* < 0.05.

### Ethical approval and participation consent.

The NHIRD in Taiwan secures patient privacy through the encryption of personal data. The data released for research purposes comprises anonymous identification numbers related to relevant claims information, such as an individual’s prescriptions, date of birth, and sex. Access to the NHIRD does not require patient consent. China Medical University’s Institutional Review Board determined that our study met the criteria for exemption (CMUH104-REC2-115-CR2); thus, informed consent was waived.

In Japan, the “Ethical Guidelines for Medical and Health Research Involving Human Subjects” do not apply to studies solely involving anonymized data and specimens that cannot be traced to individuals. Accordingly, an ethical review was not required.

## Results

### Matched cohorts

Our study population comprised individuals born in different seasons, with detailed information on these patients’ comorbidities and demographics provided in Table 1. Males and females accounted for 51% and 49% of the study population, respectively. The dominant groups in terms of age, occupation, urbanization level, and income were 20–39-year-olds (36%), office workers (56%), dwellers in areas with the highest urbanization level (30%), and individuals with the lowest income (60%), respectively. There were no significant differences in sex, age group, occupation, urbanization level, and income among people born in the different seasons (based on winter) (SMD < 0.1).

**Table 1:** Demographic and clinical characteristics of death cases including age, gender, comorbidities, and other relevant factors, categorized by the season in which they were born

Variable	Season of birth				SMD (spring vs. winter)	SMD (summer vs. winter)	SMD (fall vs. winter)
	Winter (Dec–Feb)	Spring (Mar–May)	Summer (June–Aug)	Fall (Sep–Nov)			
Sex					0.002	0.002	0.001
Female	103,701 (48.99)	103,924 (49.09)	103,506 (48.89)	103,636 (48.95)			
Male	107,996 (51.01)	107,774 (50.91)	108,195 (51.11)	108,065 (51.05)			
Age					0.012	0.015	0.012
< 20	68,416 (32.32)	68,040 (32.14)	69,605 (32.88)	69,486 (32.82)			
20–39	76,834 (36.29)	76,464 (36.12)	75,883 (35.84)	75,913 (35.86)			
40–64	51,433 (24.30)	52,502 (24.80)	50,823 (24.01)	51,122 (24.15)			
≥ 65	15,014 (7.09)	14,692 (6.94)	15,390 (7.27)	15,180 (7.17)			
Occupation					0.002	0.010	0.008
Office workers	118,415 (55.94)	118,246 (55.86)	118,323 (55.89)	118,758 (56.10)			
Manual workers	72,905 (34.44)	73,070 (34.52)	72,422 (34.21)	72,237 (34.12)			
Others	20,377 (9.63)	20,382 (9.63)	20,956 (9.90)	20,706 (9.78)			
Urbanization					0.010	0.004	0.003
I (highest)	63,598 (30.04)	63,883 (30.18)	63,692 (30.09)	63,374 (29.94)			
II	63,240 (29.87)	63,359 (29.93)	63,472 (29.98)	63,382 (29.94)			
III	38,829 (18.34)	38,072 (17.98)	38,883 (18.37)	39,038 (18.44)			
IV	46,030 (21.74)	46,384 (21.91)	45,654 (21.57)	45,907 (21.68)			
Income					0.006	0.013	0.015
0–15840	126,087 (59.56)	125,695 (59.37)	127,346 (60.15)	127,572 (60.26)			
15,841–28,800	60,976 (28.80)	61,476 (29.04)	59,886 (28.29)	59,813 (28.25)			
28,801–45800	17,167 (8.11)	17,011 (8.04)	17,004 (8.03)	16,851 (7.96)			
> 45,800	7467 (3.53)	7516 (3.55)	7465 (3.53)	7465 (3.53)			
Baseline comorbidity							
Cancer	1229 (0.58)	1302 (0.62)	1272 (0.60)	1143 (0.54)	0.004	0.003	0.005
Diabetes	9609 (4.54)	9853 (4.65)	9823 (4.64)	9386 (4.43)	0.006	0.005	0.005
Hypertension	21,147 (9.99)	21,758 (10.28)	24,576 (10.19)	20,832 (9.84)	0.010	0.007	0.005
Coronary artery disease	10,134 (4.79)	10,354 (4.89)	10,480 (4.95)	10,137 (4.79)	0.005	0.008	< 0.001
Head injury	4043 (1.91)	4143 (1.96)	4153 (1.96)	3920 (1.85)	0.003	0.004	0.004
Depression	1691 (0.80)	1794 (0.85)	1749 (0.83)	1582 (0.75)	0.005	0.003	0.006
Hyperlipidemia	12,162 (5.75)	12,507 (5.91)	12,507 (5.91)	12,181 (5.75)	0.007	0.007	< 0.001
Stroke	3873 (1.83)	4098 (1.94)	4189 (1.98)	3799 (1.79)	0.008	0.011	0.003
Mental disease	25,192 (11.90)	25,638 (12.11)	25,675 (12.13)	24,705 (11.67)	0.006	0.007	0.007
Neurologic disease	98 (0.05)	83 (0.04)	91 (0.04)	88 (0.04)	0.003	0.002	0.002
Chronic kidney disease	6935 (3.28)	7128 (3.37)	7165 (3.38)	6716 (3.17)	0.005	0.006	0.006
Dialysis	233 (0.11)	236 (0.11)	270 (0.13)	213 (0.10)	< 0.001	0.005	0.003
Allergic disease	15,327 (7.24)	15,536 (7.34)	15,573 (7.36)	15,147 (7.15)	0.004	0.004	0.003
Dementia	403 (0.19)	415 (0.20)	410 (0.19)	400 (0.19)	0.001	0.001	< 0.001
COPD	8514 (4.02)	8613 (4.07)	8484 (4.01)	8255 (3.90)	0.002	0.001	0.006
Osteoporosis	2845 (1.34)	2847 (1.34)	2872 (1.36)	2784 (1.32)	< 0.001	0.001	0.003
Sleep disorder	4907 (2.32)	5104 (2.41)	5124 (2.42)	4707 (2.22)	0.006	0.007	0.006

The birth years of these people ranged from 1900 to 2012, with the average birth year 1968.

Among the patients with comorbidities, approximately 0.6% were diagnosed with cancer, 4.5% with diabetes, 10% with hypertension, and around 5% with coronary artery disease, 2% had a head injury, 0.8% had depression, 5.8% had hyperlipidemia, approximately 2% had a stroke, 12% had mental disorders (including psychosis, other psychoses, neurotic disorders, personality disorders, and other nonpsychotic mental disorders, and mental retardation), 1.3% had osteoporosis, 0.04% had a neurologic disease, 3.3% had chronic kidney disease, 0.1% were on dialysis, 7.3% had the allergic condition, 0.2% had dementia, 4% had the COPD, and 2.4% had a sleep disorder.

### Association between all-cause mortality and the season of birth

Our results indicated a significantly higher all-cause mortality risk for individuals born in spring and summer compared to those born in winter, with the associated HRs (95% CIs) of 1.06 (1.02–1.10) and 1.04 (1.00–1.08), respectively. There was no statistically significant difference between fall and winter births, with an HR (95% CI) of 0.99 (0.95–1.03).

**Fig. 1:**
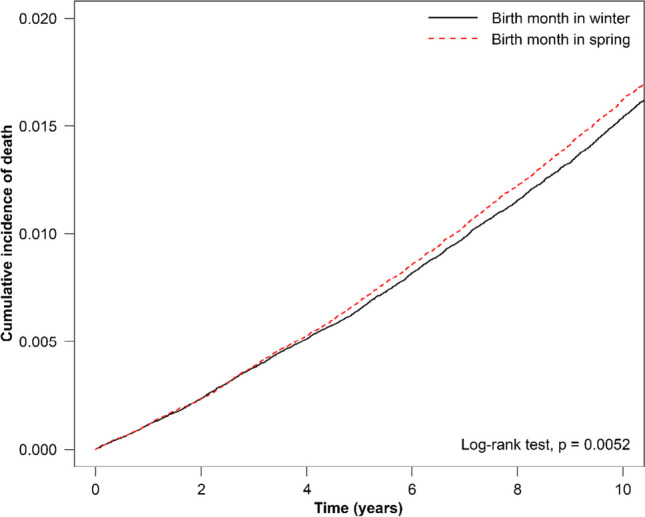
Cumulative incidence of all-cause mortality for individuals born in winter versus spring

**Fig. 2:**
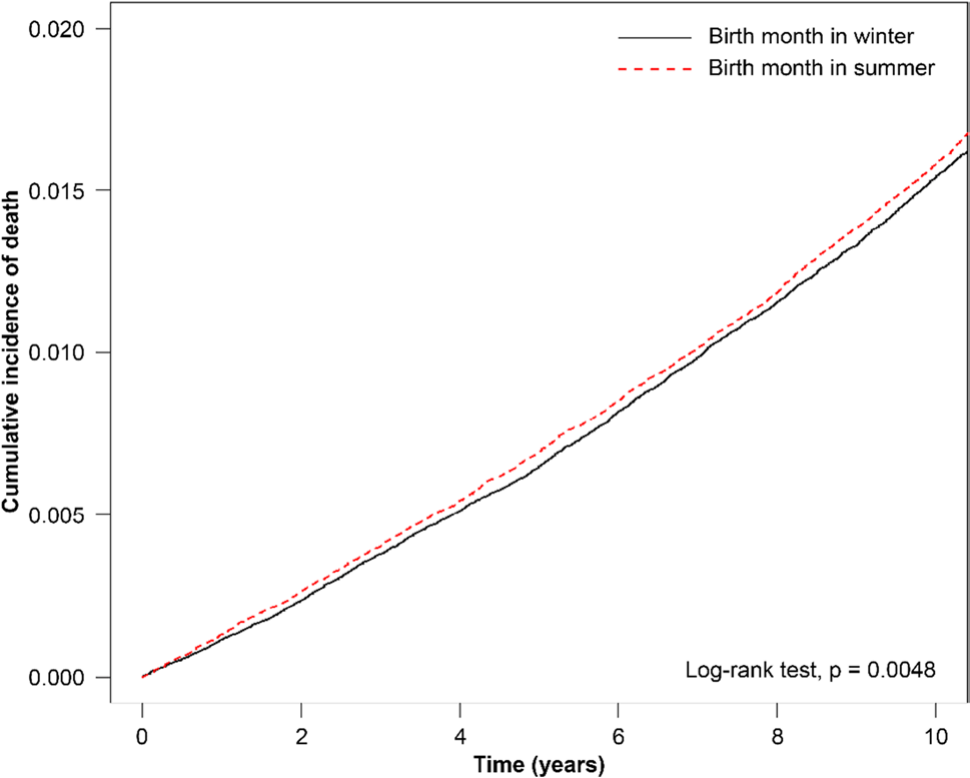
Cumulative incidence of all-cause mortality for individuals born in winter versus summer

**Fig. 3:**
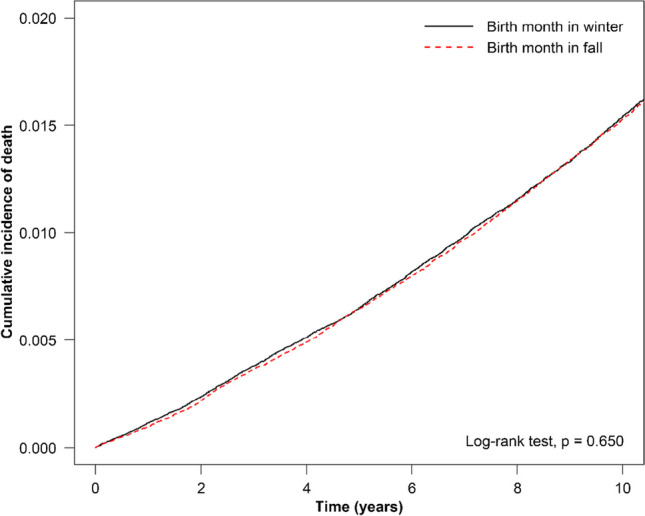
Cumulative incidence of all-cause mortality for individuals born in winter versus fall

Our results showed that the cumulative incidence of all-cause mortality was significantly higher in individuals born in spring compared to those born in winter (*p* = 0.0052; Fig. 1). In addition, we observed the cumulative incidence of death to be significantly higher among individuals born in summer than among those born in winter (*p* = 0.048, Fig. 2). Finally, we identified the cumulative incidence of all-cause mortality to not differ significantly between individuals born in winter and those born in fall (*p* = 0.650; Fig. 3). After adjusted by demographic factors and comorbidities, patients who was born in spring still had 1.05-fold risk of all-cause mortality than patients who was born in winter (adjusted HR=1.05, 95% CI = 1.01-1.09). Patients with other different stratification had no significant higher risk of all-cause mortality of different season of birth.

## Discussion

Upon adjusting for comorbidities, our examination of the correlation between the birth month and life expectancy revealed that individuals born in spring have a lower life expectancy than those born in winter. However, no difference in life expectancy was noted between autumn-born and winter-born individuals or between summer-born and winter-born individuals. Our findings align with those of previous studies which show that among adults aged 50 and above, those born in spring have a lower life expectancy than their autumn-born counterparts (Moore et al. 1997; Doblhammer and Vaupel 2001; Ueda et al. 2013). Since previous studies have reported shorter life spans for spring-born compared to other seasons, we focused on spring-born in this study as well. For example, a study by Ueda et al. reported higher mortality rates for spring and summer births (Ueda et al. 2013), which is consistent with our findings.

The fact that differences in life expectancy were observed despite adjustment for complications suggests that there may have been factors that were not fully adjusted for. For example, since it has been suggested that exposure to sunlight may induce telomere shortening (Ikeda et al. 2014), it is possible that prolonged exposure to ultraviolet light during childhood may have affected telomeres, resulting in an effect on life span. Among sunlight, Ultraviolet A-waves (UVA) are known to peak in spring and summer, and UVA has been reported to affect telomere shortening. UVA have been shown to shorten cell telomeres; in a study by Oikawa et al. (2001), UVA (365-nm) irradiation shortened telomeres in WI-38 fibroblasts, and 8-oxo-7,8-dihydro-2'-deoxyguanosine (8- oxodG) formation was shown to increase (Oikawa et al. 2001). Kawanishi and Oikawa (2004) showed that UVA irradiation induces specific DNA damage (formation of 8-oxodG) within the telomere sequence, which contributes to telomere shortening (Kawanishi and Oikawa 2004). Thus, it is possible that UVA affects telomere shortening, causing a shortened lifespan.

Previous studies have reported variances in disease incidence according to birth month (Foster and Roenneberg 2008). In particular, the incidence of psychiatric disorders and neurological illnesses is higher among spring-born and summer-born individuals (Foster and Roenneberg 2008); however, in this study, the prevalence of mental sickness tended to be higher among individuals born in spring and summer; however, the majority of neurological illnesses did not differ significantly among individuals born in different seasons. Although the incidence of asthma among spring-born and summer-born individuals was also reported to be higher (Foster and Roenneberg 2008), the prevalence of allergic diseases also tended to be higher among individuals born in spring and summer in the present study.

Vitamin D deficiency in pregnant women, which might differ by season, has been reported to increase the risk of conditions such as childhood obesity (Crozier et al. 2012). We suspect that melatonin is involved. Geomagnetic disturbance peaks in spring (Nishimura et al. 2020), and fluctuations in the magnetic field reportedly reduce melatonin secretion (Cherry 2002). It has been pointed out that decreased melatonin secretion may increase the incidence of cancer, neurological diseases, and heart diseases because melatonin has a strong antioxidant effect (Cherry 2002).

In the future, more informed decisions about preferred birth seasons may become common among prospective parents. For example, if parents have allergic diseases, giving birth in a season other than spring may be recommended.

Our study has confirmed the existence of a correlation between birth month and life expectancy after adjusting for comorbidities. Future research will need to take into account lifetime exposure to environmental factors.

## Data Availability

The data underlying this study is from the National Health Insurance Research Database, which has been transferred to the Health and Welfare Data Science Center (HWDC). Interested researchers can obtain the data through formal application to the HWDC, Department of Statistics, Ministry of Health and Welfare, Taiwan (http://dep.mohw.gov.tw/DOS/np-2497-113.html). Those interested would be able to access the data in the same manner as the authors. The authors did not have any special access privileges that others would not have.

## References

[CR1] Cherry N (2002) Schumann resonances, a plausible biophysical mechanism for the human health effects of Solar/Geomagnetic activity. Nat Hazards 26(3):279–331

[CR2] Cozzani M, Barban N et al (2023) Birth month and adult lifespan: A within-family, cohort, and spatial examination using FamiLinx data in the United States (1700–1899). Demographic Research 49(9): 201–218. 10.4054/DemRes.2023.49.9. https://www.demographic-research.org/volumes/vol49/9/files/readme.49-9.txt. https://www.demographic-research.org/volumes/vol49/9/files/demographic-research.49-9.zip

[CR3] Crozier SR, Harvey NC et al (2012) Maternal vitamin D status in pregnancy is associated with adiposity in the offspring: findings from the Southampton Women’s Survey. Am J Clin Nutr 96(1):57–63. 10.3945/ajcn.112.037473 22623747 10.3945/ajcn.112.037473 PMC4632192

[CR4] Doblhammer G, Vaupel JW (2001) Lifespan depends on month of birth. Proc Natl Acad Sci U S A 98(5):2934–2939. 10.1073/pnas.041431898 11226344 10.1073/pnas.041431898 PMC30243

[CR5] Foster RG, Roenneberg T (2008) Human responses to the geophysical daily, annual and lunar cycles. Curr Biol: CB 18(17):R784–R794. 10.1016/j.cub.2008.07.003 18786384 10.1016/j.cub.2008.07.003

[CR6] Gavrilov LA, Gavrilova NS (2011) Season of birth and exceptional longevity: comparative study of american centenarians, their siblings, and spouses. J Aging Res 2011:104616. 10.4061/2011/104616 22187646 10.4061/2011/104616 PMC3236478

[CR7] Ikeda H, Aida J et al (2014) Quantitative fluorescence in situ hybridization measurement of telomere length in skin with/without sun exposure or actinic keratosis. Hum Pathol 45(3):473–480. 10.1016/j.humpath.2013.10.009 24411948 10.1016/j.humpath.2013.10.009

[CR8] Kawanishi S, Oikawa S (2004) Mechanism of telomere shortening by oxidative stress. Ann N Y Acad Sci 1019:278–284. 10.1196/annals.1297.047 15247029 10.1196/annals.1297.047

[CR9] Lerchl A (2004) Month of birth and life expectancy: role of gender and age in a comparative approach. Naturwissenschaften 91(9):422–425. 10.1007/s00114-004-0553-5 15338031 10.1007/s00114-004-0553-5

[CR10] Moore SE, Cole TJ et al (1997) Season of birth predicts mortality in rural Gambia. Nature 388(6641):434. 10.1038/41245 9242401 10.1038/41245

[CR11] Nishimura T, Tsai IJ et al (2020) Association of Geomagnetic Disturbances and Suicide Attempts in Taiwan, 1997–2013: A Cross-Sectional Study. Int J Environ Res Public Health 17(4). 10.3390/ijerph17041154 10.3390/ijerph17041154 PMC7068248 32059562

[CR12] Oikawa S, Tada-Oikawa S et al (2001) Site-specific DNA damage at the GGG sequence by UVA involves acceleration of telomere shortening. Biochemistry 40(15):4763–4768. 10.1021/bi002721g 11294644 10.1021/bi002721g

[CR13] Ueda P, Edstedt Bonamy AK et al (2013) Month of birth and mortality in Sweden: a nation-wide population-based cohort study. PLoS ONE 8(2):e56425. 10.1371/journal.pone.0056425 23457566 10.1371/journal.pone.0056425 PMC3574007

[CR14] Vaiserman AM, Collinson AC et al (2002) Seasonal programming of adult longevity in Ukraine. Int J Biometeorol 47(1):49–52. 10.1007/s00484-002-0144-0 12461609 10.1007/s00484-002-0144-0

[CR15] Zhang Y, Devore EE et al (2019) Birth month, birth season, and overall and cardiovascular disease mortality in US women: prospective cohort study. BMJ (Clin Res Ed) 367:l6058. 10.1136/bmj.l6058 10.1136/bmj.l6058 PMC7190053 31852664

